# Delirium on stroke units: a prospective, multicentric quality-improvement project

**DOI:** 10.1007/s00415-022-11000-6

**Published:** 2022-02-14

**Authors:** Peter Nydahl, Friederike Baumgarte, Daniela Berg, Manuela Bergjan, Christoph Borzikowsky, Christiana Franke, Diana Green, Anisa Hannig, Hans Christian Hansen, Armin Hauss, Uta Hansen, Rahel Istel, Norma Krämer, Karita Krause, Renée Lohrmann, Mohammad Mohammadzadeh-Vazifeh, Jürgen Osterbrink, Frederick Palm, Telse Petersen, Bernd Schöller, Henning Stolze, Max Zilezinski, Johannes Meyne, Nils G. Margraf

**Affiliations:** 1 Nursing Science and development, Department of Anesthesiology and Intensive Care Medicine, University Hospital of Schleswig-Holstein, Kiel, Germany; 2 Christian Albrechts University Kiel, Kiel, Germany; 3 Department of Neurology, University Hospital of Schleswig-Holstein, Kiel, Germany; 4 Business Division Nursing Directorate, Nursing Science, Charité – Universitätsmedizin Berlin, corporate member of Freie Universität Berlin, Humboldt-Universität zu Berlin and Berlin Institute of Health, Berlin, Germany; 5 Institute of Medical Informatics und Statistics, University Hospital of Schleswig-Holstein, Kiel, Germany; 6 Department of Neurology, Charité – Universitätsmedizin Berlin, corporate member of Freie Universität Berlin, Humboldt-Universität zu Berlin and Berlin Institute of Health, Berlin, Germany; 7 Department of Neurology, Friedrich-Ebert-Krankenhaus, Neumünster, Germany; 8 Department of Neurology, Diako Flensburg, Flensburg, Germany; 9 Institut für Pflegewissenschaft und-praxis, Paracelsus Medizinische Privatuniversität, Salzburg, Austria; 10 Brooks College of Health, University of North Florida, Jacksonville, USA; 11 Department of Neurology, Heliosklinikum Schleswig, Schleswig, Germany; 12 University Medicine Halle (Saale), Health Service Research Working Group | Acute Care, Department of Internal Medicine, Faculty of Medicine, Martin-Luther-University Halle-Wittenberg, Halle, Germany

**Keywords:** Delirium, Encephalopathy, Quality-improvement, Stroke

## Abstract

**Background:**

Post-stroke delirium (POD) in patients on stroke units (SU) is associated with an increased risk for complications and poorer clinical outcome. The objective was to reduce the severity of POD by implementing an interprofessional delirium-management.

**Methods:**

Multicentric quality-improvement project on five SU implementing a delirium-management with pre/post-comparison. Primary outcome was severity of POD, assessed with the Nursing Delirium Screening Scale (Nu-DESC). Secondary outcome parameters were POD incidence, duration, modified Rankin Scale (mRS), length of stay in SU and hospital, mortality, and others.

**Results:**

Out of a total of 799 patients, 59.4% (*n* = 475) could be included with 9.5% (*n* = 45) being delirious. Implementation of a delirium-management led to reduced POD severity; Nu-DESC median: pre: 3.5 (interquartile range 2.6–4.7) vs. post 3.0 (2.2–4.0), albeit not significant (*p* = 0.154). Other outcome parameters were not meaningful different. In the post-period, delirium-management could be delivered to 75% (*n* = 18) of delirious patients, and only 24 (53.3%) of delirious patients required pharmacological treatments. Patients with a more severe stroke and POD remained on their disability levels, compared to similar affected, non-delirious patients who improved.

**Conclusions:**

Implementation of delirium-management on SU is feasible and can be delivered to most patients, but with limited effects. Nursing interventions as first choice could be delivered to the majority of patients, and only the half required pharmacological treatments. Delirium-management may lead to reduced severity of POD but had only partial effects on duration of POD or length of stay. POD hampers rehabilitation, especially in patients with more severe stroke.

**Registry:**

DRKS, DRKS00021436. Registered 04/17/2020, www.drks.de/DRKS00021436.

**Supplementary Information:**

The online version contains supplementary material available at 10.1007/s00415-022-11000-6.

## Background

Delirium is a neuropsychiatric syndrome, based on acute encephalopathy, and a common complication in patients on Stroke Units (SU) [1–3]. Delirium is characterized by disorders of attention and concentration, rapid development and fluctuation during the day, and additional cognitive disorders [4]. Delirium is a direct result of one or more physical disorders, interventions or medications [5]. The causes of delirium are manifold and result from predisposing and triggering factors [6–9]. Consequences are an increased risk of prolonged ventilation and length of stay in SU and hospital, increased mortality, permanent cognitive disturbances and institutionalization [10]. The incidence of delirium in patients in intensive care and intermediate care units varies between 20 and 89% [11], in stroke patients in 25% [12], in recent studies about 16% [12].

Based on clinical experience, various problems in delirium-management in our SU have been identified: (a) there are no in-house, general recommendations for the treatment of delirium, (b) the causes of delirium are insufficiently identified and treated in everyday clinical practice, (c) the treatment of delirium on SU is usually carried out at the discretion and experience of the responsible physician and most times primarily pharmacologically, (d) junior physicians often order non-specific standard medications, (e) the concepts often change with the responsible physicians and often between day and night services, (f) due to staff shortages, nurses can only insufficiently provide care for delirious patients and therefore favor pharmacological and non-pharmacological restraints, (g) about one-third of delirious patients are transferred to subsequent wards, with hardly any recommendations given for the continuation, adaptation or discontinuation of pharmacological delirium treatment.

These local problems in the care of delirious patients are not uncommon. Surveys in delirium-management showed that less than the half of clinicians have standardized delirium-management, and less than one-third are using valid assessment frequently [13, 14]. Pharmacological therapies were chosen much more frequently in the setting of an intensive care unit, the most frequently mentioned were Haloperidol, Clonidine, and Melperone. The main barriers for implementing delirium-management are the lack of knowledge, training, and interprofessional communication and cooperation [15–18]. A standardized delirium-management, which includes both pharmacological and non-pharmacological measures, could help to avoid these problems [10, 19]. A recent guideline for acute stroke recommends frequent delirium assessment and management [20], and a quality-improvement project was required in advance [21]. Frontline nurses of the involved SU suggested to perform a quality-improvement project. Hence, the objective of this quality-improvement project was to reduce the severity of delirium in stroke patients by implementing an interprofessional delirium-management and to estimate its effect size.

## Methods

The study has been approved by local ethic committees (D459/20) and registered (DRKS00021436). The report of this quality-improvement project is based on the criteria of the revised Standards for Quality-improvement Reporting Excellence SQUIRE (see Supplement Table E7) [22]. Data are available for reasonable request.

### Design

**Fig. 1:**
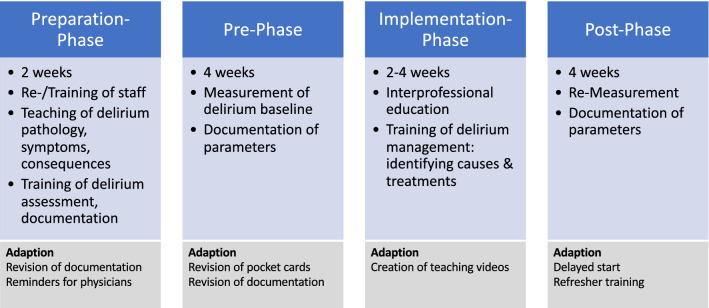
Improvement process and adaptions

A non-blinded, prospective, exploratory, multicentric quality-improvement project with pre/post-comparison was conducted, planned for 10 weeks. Participating clinicians received a re-/training for delirium assessment and implemented a standardized delirium-management, resulting in four phases: (1) delirium assessment (2 weeks), (2) pre-implementation phase, measuring delirium baseline (4 weeks), (3) education and implementation phase (4 weeks), (4) post-implementation phase, measuring delirium improvement (4 weeks). Phase (2) and (4) were compared to assess the impact of the delirium-management (Fig. 1).

The delirium-management included: (a) delirium screening three times within 24 h, using the Nursing Delirium Screening Scale (Nu-DESC); (b) in case of positive results, validation by treating physician using the Diagnostic and Statistical Manual of Mental Disorders, Version V (DSM-V) criteria [5]; (c) interprofessional evaluation of possible reasons and treatment of underlying causes using checklists (Table E1); (d) non-pharmacological interventions such as re-orientation, education, mobilization, and others (e) in case of persisting symptoms, specific pharmacological interventions; and (f) report of complications, such as reduced compliance, sedation, immobility, and others.

### Education

All participating centers received a training in delirium assessment. The interprofessional training took place several times in each center for 30 min. Due to the second wave of the COVID crises, educational meetings on SU were changed to virtual meetings. Pocket cards, posters, and videos were issued for support, especially for all frontline nurses. The introduction of the delirium-management was standardized by the study team in multiple 30-min training courses, which were carried out on the SU, resp. in online-meetings. The education was complemented by teaching videos, pocket cards for the staff, and posters per patient and treatment room. Documentation was adapted to the management.

### Setting

In total, 6 SU in 5 hospitals participated in the study, covering 53 beds.

### Study population

Patients were screened at admission on SU for recruitment. Inclusion criteria were: (a) aged ≥ 18 years, (b) suspected stroke, and (c) consent by themselves or legal representative to use their data for research. Exclusion criteria were: (a) being in a hospital > 24 h prior to admission on SU, (b) severe disturbances of consciousness and not assessable for delirium screening, (c) high probability of death within 48 h, (d) not assessable for delirium assessment (deaf, total aphasia, foreign language, and others [23].

### Outcome variables

Local study coordinators extracted all data from patients’ charts. Sociodemographic data of included patients were collected from charts, such as categorized age (in percentiles < 20 years, < 30 years, … < 110 years), gender, living at home vs. nursing facility, known depression, dementia, body mass index, and others (Table 1).

**Table 1:** Sociodemographic data

Item^a^	All (*n* = 475)	Pre-implementation (*n* = 251)	Post-implementation (*n* = 224)	*p*^b^
Preadmission				
Most frequent age decile in years (%)	< 80 (28.5)	< 80 (28.8)	< 80 (28.2)	0.398
Female gender	194 (43.6)	109 (45.6)	85 (41.3)	0.389
Living at home	422 (95)	228 (95.4)	194 (94.6)	0.827
Living in nursing home	22 (5)	11 (4.6)	11 (5.4)	
Pre-existing depression	11 (2.3)	4 (1.6)	7 (3.1)	0.192
Pre-existing dementia	16 (3.4)	10 (4)	6 (2.7)	0.529
Modified Rankin Scale	0 (0–1)	0 (0–1)	0 (0–0)	0.009^ns^
Admission				
C-reactive protein (mg/l)	2.5 (1–6)	2.3 (1–6.5)	2.7 (1–6)	0.744
Natrium (mmol/l)	139 (137–141)	139 (137–141)	139 (137–141)	0.827
Body Mass Index (kg/m^2^)	26.2 (23.6–29.4)	25.9 (23.4–29.4)	26.3 (23.6–29.3)	0.661
Neurological state				
NIH-SS	2 (0–4)	1 (0–4)	2 (0–4)	0.660
Modified Rankin Scale	2 (1–3)	2 (1–4)	2 (1–3)	0.295
Primary diagnosis				0.101
Ischemic stroke	319 (69.5)	176 (71.8)	143 (66.8)	
Trans ischemic attack	93 (20.3)	51 (20.8)	42 (19.6)	
Hemorrhagic stroke	13 (2.8)	4 (1.6)	9 (4.2)	
Cerebral venous sinus thrombosis	1 (0.2)	0 (0)	1 (0.2)	
Epilepsy	3 (0.7)	0 (0)	3 (0.7)	
Migraine	4 (0.9)	2 (0.8)	2 (0.9)	
Others	26 (5.7)	9 (3.7)	17 (7.9)	
Interventions				
Intravenous thrombolysis	47 (9.9)	24 (9.6)	23 (10.3)	0.878
Endovascular thrombectomy	22 (4.6)	13 (5.2)	9 (4)	0.663
Anticholinergic Risk Scale^c^	73	29	44	0.794

*NIH-SS* National Institutes of Health Stroke Scale, *ns* not significant

^a^Data are reported as number (percent), resp. median (Interquartile Range). Percentages may not sum up to 100 due to rounding

^b^To avoid false-positive results by multiple testing, the *p* level is adjusted to *p* = 0.0005

^c^In total, 46 patients received anticholinergic drugs (pre-phase: *n* = 22, post-phase: *n* = 24)

Before and during the stay on SU, treatment-specific data were collected, such as primary admission diagnosis, interventions as intravenous thrombolysis and/or endovascular thrombectomy, National Institutes of Health Stroke Scale (NIH-SS) at admission categorized into NIH-SS = 0 (no stroke symptoms), NIH-SS = 1–4 (minor symptoms), NIH-SS = 5–42 (moderate to severe stroke), modified Rankin Scale (mRS), laboratory data (C-reactive protein, Natrium) and anticholinergic medications using the Anticholinergic Risk Scale [24] (Table 1).

### Primary outcome

Primary outcome parameter is the severity of delirium, assessed with the Nursing Delirium Screening Scale (Nu-DESC), a valid, reliable instrument for delirium detection [25–27]. The Nu-DESC has five dimensions with point values of 0 = non-existent, 1 = present and 2 = strongly present, the probability of delirium is given from a sum point value ≥ 2. It can thus be used as a metric scale of 0–10 with 10 = most severe delirium; delirious patients were assessed with a mean value of 7 [28]. The Nu-DESC is available in German translation [29] and has been validated for use in Intermediate Care (IMC) patients including stroke patients [30–32]. The Nu-DESC was collected and documented by nurses at the bedside three times within 24 h during the study. In the case of a first positive screening, the responsible physician was informed and validated the findings using the DSM-V criteria [5]. To calculate the severity of delirium, only Nu-DESC scores during delirious episodes were used. According to our registered protocol, the use and diagnostic value of an additional test for attention has been analyzed and will be reported later.

### Secondary outcome parameters

Secondary outcome parameters were presence, type, and duration of delirium. The end of delirium was defined as last positive screening, if patients were free of delirium symptoms for 24 h, or were discharged from SU. Further parameters were calculation of the effect size, mortality in SU and hospital, length of stay in SU and hospital, mRS at discharge from SU, severe disability (mRS 3–6), and early rehabilitation on SU (difference between mRS_Admission_ and mRS_Discharge_). Delirium-management-related parameters were (a) number of non-pharmacological interventions performed by frontline nurses, therapists, and physicians, such as verbal re-orientation, providing a day-night rhythm, out-of-bed mobilization between 6.00 a.m. and 11.00 p.m., description of delirium-related symptoms (verbalization of hallucinations, sleep disorders, disattention, and comfort), supporting in eating and drinking, education about delirium (risk factors, symptoms, and consequences), engagement for collaboration (patient calls clinician if he/she perceives delirium symptoms), cognitive stimulation (newspapers, television), provision of hearing/vision aids, education of family (what is delirium, how to help, provision of leaflets), extended visiting times in case of delirium, protected environment in case of hyperactive delirium (single bed room, earplugs); (b) pharmacological, symptom-specific interventions such as (1) agitation: melperone, clonidine, pipamperone, quetiapine, (2) vegetative symptoms: clonidine, dexmedetomidin, (3) psychotic symptoms: haloperidol, quetiapine, risperidone, (4) anxiety symptoms: lorazepam, diazepam, and (5) sleep disorders: melatonin, melperone; (c) identified precipitants and treatments for delirious patients (Table E1); (d) complications, defined as presence of dehydration, undernutrition, pressure sore, fall, immobility, restraints, decreasing compliance, or removal of lines. Outcomes such as study performance, 90-day outcome and others will be published later.

### Power calculation

Based on usual bed occupancy, 495 patients would have been admitted to all participating SU per month, giving 990 patients in total. Based on in- and exclusion criteria, 70% (*n* = 693) of patients could have been recruited and included in the study [33]. The incidence of delirium was estimated with 16% (*n* = 110 with *n* = 55 in each group) [12]. Estimating a mean Nu-DESC of 7 points (Standard deviation of ± 2) in delirious patients and an alpha of 5%, the study would have a power of 74.6% to prove a pre/post-difference of 1 point; in case of 2 points decrease, the power would have increased to 99.9% [34].

### Statistical analysis

The analysis of nominal data is reported as absolute and relative frequencies (percentages), ordinal data in its modus. Due to non-normal distribution in most outcome variables, metrical data are reported as medians and Interquartile Ranges (IQRs). Inferential tests were conducted using Fisher’s Exact test, Chi-squared test, Wilcoxon test, Kruskal–Wallis test, and for correlation analyses, Spearman’s rho. Logistic regression was planned to adjust for confounders, if the number of participants was at least ≥ 10 per confounder. In total, we conducted 92 tests to prove significant relationships between interesting variables; to avoid false-positive results, the level of significance has been reduced by a conservative Bonferroni correction to (*p*_adjust_ = 0.05/*n*_tests_) *p*_adjust_ = 0.000543. The analysis is carried out with SPSS 23 (IBM, New York).

## Results

All participating SU implemented delirium assessment, measured the baseline in the pre-phase, implemented the delirium-management, and measured the improvement in the post-phase. During this process, several adaptions were required, such as a revision of documentation due to higher efficiency, or pocket cards due to more different than expected workflows in practice (Fig. 1). Only two centers conducted the study within the planned time frame of close to 3 months, three centers required in mean the threefold duration due to the pandemic and delayed start of the pre- and/or post-period. Most important adaptions were delayed starting points of the post-phase in three centers due to higher workload of local researchers during COVID crisis, requiring creation of teaching videos instead of live-teachings, and refresher trainings before the beginning of the post-phase.

Overall, 799 patients were admitted to participating SU and screened for inclusion, and 475 (59.4%) could be included in the study: 52.8% (*n* = 251) in the pre-phase, 47.1% (*n* = 224) in the post-phase. There were neither significant differences in socio-demographic and admission data of included patients between the pre- vs. post-phase (Table 1) nor between different centers, except for difference between centers in the modified Rankin Scale at admission (Supplement Table E2). Delirium screening rate, assessed in three centers, was in median 89.6% (IQR 62.5–100%) with no significant differences between the pre/post-phase [80% (IQR 60–100%) vs. 100% (IQR 66.7–100%); *p* = 0.01].

The primary outcome Nu-DESC was reduced by half a point after implementation of delirium-management [pre: 3.5 (IQR 2.6–4.7) vs. post 3.0 (2.2–4.0), albeit not significant; *p* = 0.154]. The effect size for reducing delirium in stroke patients by implementation of a delirium-management was *d* = − 0.205 (*p* = 0.177).

In total, 9.5% (*n* = 45) patients were delirious (pre: 8.4% (*n* = 21) vs. post: 10.7% (*n* = 24), *p* = 0.434). Delirious patients were more likely living in a nursing home beforehand and had a more severe stroke, compared to non-delirious patients (Table E3). Adjustment of confounders was not feasible due to a low number of delirious patients per confounder.

Implementation of delirium-management had no significant effects on other secondary outcome parameters in delirious patients such as incidence and type of delirium, length of stay, discharge destination, and others (Table 2).

**Table 2:** Delirium-related data in delirious patients

Item^a^	All (*n* = 45)	Pre-implementation (*n* = 21)	Post-implementation (*n* = 24)	*p*^b^
Type of delirium				0.069
Hyperactive delirium	6 (13.3)	1 (4.8)	5 (20.8)	
Hypoactive delirium	5 (11.1)	2 (9.5)	3 (12.5)	
Mixed delirium	9 (20)	2 (9.5)	7 (29.2)	
Not specified	25 (53.3)	16 (76.2)	9 (37.5)	
Days in delirium	3 (2–5)	3 (1.5–5)	3 (2–5)	0.991
Nu-DESC highest	4 (3–7)	5 (3–7)	4 (3–6.7)	0.307
Nu-DESC median	3.2 (2.3–4.3)	3.5 (2.6–4.7)	3 (2.2–4)	0.154
Patients with ≥ 1 complication	19 (42.2)	6 (28.6)	13 (54.2)	0.131
Modified Rankin Scale at discharge	4 (3–5)	4 (3–5)	4 (2–5)	0.627
Length of stay in Stroke Unit (days)	5 (3.5–7)	5 (3–8)	5 (4–7)	0.881
Length of stay in hospital (days)	11 (5.7–14.2)	9 (4–14)	11 (6–15)	0.396
Mortality in Stroke Unit	2 (4.4)	1 (4.8)	1 (4.2)	1.000
Mortality in hospital	4 (8.8)	2 (9.5)	2 (8.3)	1.000
Discharge localization				
Home	9 (20)	5 (23.8)	4 (16.7)	0.753
Rehabilitation facility	10 (22.2)	4 (19)	6 (25)	
Other hospital	7 (15.6)	4 (19)	3 (12.5)	
Nursing home	6 (13.3)	2 (9.5)	4 (16.7)	
Missing information	13 (28.9)	6 (28.6)	7 (29.2)	

*Nu-DESC* Nursing Delirium Screening Scale

^a^Data are reported as number (percent), resp. median (interquartile range). Percentages may not sum up to 100 due to rounding

^b^To avoid false-positive results by multiple testing, the *p* level is adjusted to *p* = 0.0005

^c^Complications were defined as presence of dehydration, undernutrition, pressure sore, fall, immobility, restraints, decreasing compliance, or removal of lines

Delirious patients received as first-line treatments nursing interventions, at least once reported in 80.9% (*n* = 17) vs. 91.7% (*n* = 22) in the pre- vs. post-phase (*p* = 0.828). The daily amounts of these interventions were mostly re-orientation in median 0.7 (0.5–1.0) times per day and patient, followed by provision of day-night-rhythm 0.5 (0.3–0.8), and mobilization 0.5 (0.3–1.0), without significant differences between the pre- vs. post-phase (Table 3). Only 24 (53.3) of delirious patients required pharmacological treatments, without significant differences between the pre- vs. post-phase (Table 3).

**Table 3:** Delirium-related treatments in delirious patients

Item	All (*n* = 45) *N*: median (IQR)*	Pre-implementation (*n* = 21)	Post-implementation (*n* = 24)	*p*
Nursing interventions per day				
Re-orientation	39: 0.7 (0.5–1)	17: 0.7 (0.4–0.9)	22: 0.7 (0.5–1)	0.520
Day–night rhythm	35: 0.5 (0.3–0.8)	15: 0.5 (0.2–0.7)	20: 0.6 (0.3–1)	0.441
Mobilization	32: 0.5 (0.3–1)	14: 0.5 (0.2–0.8)	18: 0.7 (0.4–1)	0.350
Description of symptoms	28: 0.4 (0.2–0.7)	12: 0.2 (0.1–0.6)	16: 0.5 (0.3–0.8)	0.036
Eating and drinking	28: 0.6 (0.3–0.8)	10: 0.6 (0.2–0.8)	18: 0.5 (0.4–1)	0.531
Education about delirium	27: 0.4 (0.3–0.6)	11: 0.3 (0.2–0.4)	16: 0.5 (0.3–0.7)	0.012
Cognitive stimulation	23: 0.4 (0.2–0.5)	7: 0.3 (0.1–0.8)	16: 0.4 (0.3–0.5)	0.482
Hearing/vision aids	18: 0.5 (0.2–1)	7: 0.3 (0.2–0.7)	11: 0.6 (0.3–1)	0.132
Education family	6: 0.2 (0.1–0.2)	3: 0.2 (0.2–0.2)	3: 0.1 (0.1–0.1)	0.513
Extended visiting times	6: 0.1 (0.1–2)	4: 0.1 (0.1–0.3)	2: 0.5 (0.1–0.5)	0.643
Protected environment	4: 0.2 (0.1–0.2)	2: 0.1 (0.1–0.1)	2: 0.2 (0.2–0.2)	0.121
Pharmacological interventions				
Patients, receiving delirium-related drugs	24 (53.3)	8 (38.1)	13 (54.2)	0.373
Total pharmacological interventions, given at least once				
Melperone	16	8	8	0.765
Pipamperone	5	3	2	0.652
Clonidine	4	2	2	1.000
Lorazepam	4	3	1	0.326
Risperidone	3	2	1	0.591
Diazepam	3	1	2	1.000
Haloperidol	2	1	1	1.000
Melatonin	2	0	2	0.491
Quetiapine	1	1	0	0.467
Dexmedetomidine	0 (0)	0	0	–

*Example: of 45 patients, 39 received at least once re-orientation, in median 0.7 (interquartile range 0.5–1) times per day

*IQR* interquartile range

Using the delirium-management in delirious patients (*n* = 24) in the post-phase, clinicians identified in 75% (*n* = 18) of patients precipitants for developing delirium and delivered specific treatments (Table E1). In these 18 patients, 96 different potential reasons for delirium were identified, of which 67.7% (*n* = 65) were treated. Nu-DESC was not significantly different in patients with delirium-management and treatment of causes vs. no management and no treatment [3 (2.2–4.8) vs. 3 (2.5–3.6); *p* = 0.887].

In patients with severe disability at admission (mRS ≥ 3), early rehabilitation on SU was lower in patients with delirium, compared to similar patients without delirium (mRS Difference_Admission-Discharge_: 0 (− 0.75 to 0.75) vs. 1 (0–2); *p* = 0.0002; Figure E1).

In general, delirious patients had a worse outcome, compared to non-delirious patients (Table E4), with differences between centers (Table E5), but not in primary outcome delirium severity (Table E6).

Based on a post hoc calculation, 608 delirious patients should have been recruited to gain significant results with 80% power and an alpha-level of 0.05.

## Discussion

In this prospective, exploratory quality-improvement project on 5 SU, using a pre/post-comparison of a before and after implementation-period of a delirium-management, 475 patients could be recruited, with nearly 10% being delirious. The implementation of a delirium-management by frontline nurses, therapists, and physicians could be delivered to 75% of delirious patients and led to a decrease of delirium burden, albeit not significant. Other parameters were similar in the pre- and post-period. Stroke patients with delirium had more severe stroke-related disabilities at admission and did not improve during their stay on SU, compared to non-delirious patients.

The implementation of a delirium-management did not reduce delirium severity to the expected extent. Recent reviews found multi-component interventions general effective in reducing delirium incidence but found limited effects on delirium severity [35, 36]. Several explanations can be discussed. In the current study, the preventive interventions in the pre-period were already at a high level, making it difficult to find a difference. The delivery of delirium-preventive nursing care increased from pre- to post-period, but if the control group is already strong, it would need very large groups to identify significant effects, what happened also in other comparable projects [37, 38]. Contrary, we still do not know the required extent of how much non-pharmacological care must be delivered to prevent patients from delirium. Rice et al. [38] implemented the Hospital Elder Life Program on SU and delivered preventive delirium care for 15 min twice a day, without significant effects. Hence, it might be questionable if the right dosage has been delivered in the present study, or if it was still too less. The main difference in implementing a delirium-management was the feature of identifying possible reasons for delirium and related treatments. Delirium is a syndrome, and it is still unknown what the most likely reasons are; Girard et al. [9] identified four causes related to critical illness responsible for 80% of ICU delirium, but in our study, these possible causes were attributed to only 9% of reasons. So far, the knowledge about the composition of possible reasons and its weighting is limited [39]. It may be likely that a stroke itself leads to inevitable disturbances in cerebral neurotransmitters and a disturbance of cerebral networking, with more severe stroke leading to a higher risk of delirium [40]. Hence, the question raises if delirium prevention might be useless to some extent in patients with stroke. Contrary, only 15% of clinicians identified stroke as reason for delirium, a fact that requires further exploration. Mobilization as intervention for prevention and treatment of delirium was often used and is recommended [41], but it remains questionable if too early and/or extended mobilization may even worsen delirium severity, as proved for the neurological outcome by too early mobilization in stroke patients [42]. At least, methodological aspects might have contributed too, such as a too short time frame of 1 month for implementation or a less outcome-sensitive screening instrument. More research about delirium, cerebral (re-)perfusion, and especially in delirium treatment by mobilization and prolonged upright positions are required to explore the processes leading to delirium in stroke patients.

In other outcome parameters, delirium is associated with an extended length of stay in SU and hospital. This is comparable and in line with recent reviews and meta-analyses, in patients with stroke and other populations [12, 43, 44]. What is new and subject of concern is the fact that those patients with more severe stroke and delirium had nearly no improvement in rehabilitation during their stay on SU, compared to similar affected patients, but without delirium. Delirium seems to hamper rehabilitation. This might be related to the core feature of delirium, inattention. Inattention might lead to less learning results how to improve movement, swallowing, or speaking after stroke again and hence, less effective rehabilitation by nurses and therapists. The ability to understand and/or produce language is essential for rehabilitation and can be reduced in stroke patients, but also in patients with delirium [45], contributing also to less rehabilitation. Both effects, combined with reduced cognition after delirium, might lead to a worse outcome for delirious stroke patients [12, 43]. More research is needed to explore how to adapt rehabilitation to delirious patients to improve the outcome in this highly vulnerable population.

This work has several main limitations. First, the targeted sample size has not been reached, most likely to reduced admission rates due to the pandemic [46] and had a lower than expected delirium incidence [12]; contrary, our targeted sample sizes would probably not have changed main outcomes and the effect can be neglected. Second, identification of delirium in stroke patients is challenging, due to conflicting neurological symptoms in stroke patients; contrary, the used screening instrument has a sufficient quality [32] and all involved clinicians were trained in its use and the biasing effect might be less severe. Third, delirium prevention by relatives, mostly effective [47], was not feasible as expected, most likely due to pandemic and visitors restrictions, and the lesser impact on the delirium-treating effect remains unknown. Fourth, solid adjustment for potential confounders was not feasible due to a low number of delirious patients. Last, the pandemic led to several adaptions, as shown in Fig. 1, extended duration of the study, and higher workload in involved clinicians; these factors might have decreased the effect size as well, but the extent cannot be estimated.

## Conclusions

The implementation of a delirium-management for stroke patients was feasible und could be delivered to 80% of included patients. The causes of delirium in stroke patients seem to be different from causes in other populations and might be more difficult to treat. Hence, optional interventions for delirium prevention and treatment might be limited. Delirium-management may lead to reduced severity of delirium but had only partial effects on duration of delirium or length of stay in SU or hospital. Delirium hampers rehabilitation, especially in patients with more severe stroke, and specific interventions to provide delirium-related rehabilitation should be developed.

## Supplementary Information

Below is the link to the electronic supplementary material.

Supplementary file1 (DOCX 69 KB)

## References

[1] Wilson JE, Mart MF, Cunningham C, Shehabi Y, Girard TD, MacLullich AMJ (2020). Delirium. Nat Rev Dis Primers.

[2] Caeiro L, Ferro JM, Albuquerque R, Figueira ML (2004). Delirium in the first days of acute stroke. J Neurol.

[3] Carin-Levy G, Mead GE, Nicol K, Rush R, van Wijck F (2012). Delirium in acute stroke: screening tools, incidence rates and predictors: a systematic review. J Neurol.

[4] Stollings JL, Kotfis K, Chanques G, Pun BT, Pandharipande PP, Ely EW (2021). Delirium in critical illness: clinical manifestations, outcomes, and management. Intensive Care Med.

[5] American-Psychiatric-Association (2013). Diagnostic and statistical manual of mental disorders.

[6] Maldonado JR (2008). Pathoetiological model of delirium: a comprehensive understanding of the neurobiology of delirium and an evidence-based approach to prevention and treatment. Crit Care Clin.

[7] Müllges W (2014). Ätiologie und therapie des delirs. Aktuelle Neurologie.

[8] Nydahl P, Margraf NG, Ewers A (2017) Delirium in stroke patients: critical analysis of statistical procedures for the identification of risk factors. Med Klin Intensivmed Notfmed 10.1007/s00063-016-0257-6 28144726

[9] Girard TD, Thompson JL, Pandharipande PP, Brummel NE, Jackson JC, Patel MB (2018). Clinical phenotypes of delirium during critical illness and severity of subsequent long-term cognitive impairment: a prospective cohort study. Lancet Respir Med.

[10] Oh ES, Fong TG, Hshieh TT, Inouye SK (2017). Delirium in older persons: advances in diagnosis and treatment. JAMA.

[11] Svenningsen H, Egerod I, Videbech P, Christensen D, Frydenberg M, Tønnesen EK (2013). Fluctuations in sedation levels may contribute to delirium in ICU patients. Acta Anaesthesiol Scand.

[12] Shaw RC, Walker G, Elliott E, Quinn TJ (2019). Occurrence rate of delirium in acute stroke settings: systematic review and meta-analysis. Stroke.

[13] Nydahl P, Dewes M, Dubb R, Hermes C, Kaltwasser A, Krotsetis S (2017). Survey among critical care nurses and physicians about delirium management. Nurs Crit Care.

[14] Krotsetis S, Nydahl P, Dubb R, Hermes C, Kaltwasser A, von Haken R (2017). Status quo of delirium management in German-speaking countries: comparison between intensive care units and wards. Intensive Care Med.

[15] Morandi A, Davis D, Taylor JK, Bellelli G, Olofsson B, Kreisel S (2013). Consensus and variations in opinions on delirium care: a survey of European delirium specialists. Int Psychogeriatr.

[16] Luetz A, Balzer F, Radtke FM, Jones C, Citerio G, Walder B (2014). Delirium, sedation and analgesia in the intensive care unit: a multinational, two-part survey among intensivists. PLoS ONE.

[17] Saller T, Dossow V, Hofmann-Kiefer K (2016). Knowledge and implementation of the S3 guideline on delirium management in Germany. Anaesthesist.

[18] Trogrlić Z, Ista E, Ponssen HH, Schoonderbeek JF, Schreiner F, Verbrugge SJ (2016). Attitudes, knowledge and practices concerning delirium: a survey among intensive care unit professionals. Nurs Crit Care.

[19] Hshieh TT, Yue J, Oh E, Puelle M, Dowal S, Travison T (2015). Effectiveness of multicomponent nonpharmacological delirium interventions: a meta-analysis. JAMA Intern Med.

[20] Ringleb PA, Hametner C, Köhrmann M, Frank B, Jansen O (2021) Acut therapy of ischemic stroke. S2-guideline, 2021. In: German Society for Neurology (ed), guidelines for diagnostics and therapies in neurology. https://www.awmf.org/uploads/tx_szleitlinien/030-046l_S2e_Akuttherapie-des-ischaemischen-Schlaganfalls_2021-05.pdf.

[21] Lele AV, Moheet AM (2020). Neurocritical care quality improvement practices: a survey of members of the Neurocritical Care Society. Neurocrit Care.

[22] Ogrinc G, Davies L, Goodman D, Batalden P, Davidoff F, Stevens D (2016). SQUIRE 2.0 (Standards for QUality Improvement Reporting Excellence): revised publication guidelines from a detailed consensus process. BMJ Qual Saf.

[23] Weiss B, Paul N, Spies CD, Ullrich D, Ansorge I, Salih F et al. (2021) Influence of patient-specific covariates on test validity of two delirium screening instruments in neurocritical care patients (DEMON-ICU). Neurocrit Care, pp 1–11 10.1007/s12028-021-01319-9 PMC8351768 34374001

[24] Rudolph JL, Salow MJ, Angelini MC, McGlinchey RE (2008). The anticholinergic risk scale and anticholinergic adverse effects in older persons. Arch Intern Med.

[25] Gusmao-Flores D, Salluh JIF, Chalhub RÁ, Quarantini LC (2012). The confusion assessment method for the intensive care unit (CAM-ICU) and intensive care delirium screening checklist (ICDSC) for the diagnosis of delirium: a systematic review and meta-analysis of clinical studies. Crit Care.

[26] van Velthuijsen EL, Zwakhalen SM, Warnier RM, Mulder WJ, Verhey FR, Kempen GI (2016). Psychometric properties and feasibility of instruments for the detection of delirium in older hospitalized patients: a systematic review. Int J Geriatr Psychiatry.

[27] Jones RN, Cizginer S, Pavlech L, Albuquerque A, Daiello LA, Dharmarajan K (2019). Assessment of instruments for measurement of delirium severity: a systematic review. JAMA Intern Med.

[28] Cinar F, Eti AF (2019). Evaluation of postoperative delirium: validity and reliability of the nursing delirium screening scale in the Turkish Language. Dement Geriatr Cogn Dis Extra.

[29] Lutz A, Radtke FM, Franck M, Seeling M, Gaudreau JD, Kleinwachter R (2008). The Nursing Delirium Screening Scale (NU-DESC). Anasthesiologie, Intensivmedizin, Notfallmedizin, Schmerztherapie AINS.

[30] Radtke FM, Franck M, Schneider M, Luetz A, Seeling M, Heinz A (2008). Comparison of three scores to screen for delirium in the recovery room. Br J Anaesth.

[31] Hargrave A, Bastiaens J, Bourgeois JA, Neuhaus J, Josephson SA, Chinn J (2017). Validation of a nurse-based delirium-screening tool for hospitalized patients. Psychosomatics.

[32] Bergjan M, Zilezinski M, Schwalbach T, Franke C, Erdur H, Audebert HJ (2020). Validation of two nurse-based screening tools for delirium in elderly patients in general medical wards. BMC Nurs.

[33] Nydahl P, Bartoszek G, Binder A, Paschen L, Margraf NG, Witt K (2017). Prevalence for delirium in stroke patients: a prospective controlled study. Brain Behav.

[34] Chow S, Shao J, Wang H (2008). Sample size calculations in clinical research.

[35] Ludolph P, Stoffers-Winterling J, Kunzler AM, Rösch R, Geschke K, Vahl CF (2020). Non-pharmacologic multicomponent interventions preventing delirium in hospitalized people. J Am Geriatr Soc.

[36] Sahawneh F, Boss L (2021). Non-pharmacologic interventions for the prevention of delirium in the intensive care unit: an integrative review. Nurs Crit Care.

[37] Rood PJ, Zegers M, Ramnarain D, Koopmans M, Klarenbeek T, Ewalds E (2021). The impact of nursing delirium preventive interventions in the intensive care unit: a multicenter cluster randomized controlled trial. Am J Respir Crit Care Med.

[38] Rice KL, Bennett MJ, Berger L, Jennings B, Eckhardt L, Fabré-LaCoste N (2017). A pilot randomized controlled trial of the feasibility of a multicomponent delirium prevention intervention versus usual care in acute stroke. J Cardiovasc Nurs.

[39] Smith M, Meyfroidt G (2017). Critical illness: the brain is always in the line of fire. Intensive Care Med.

[40] Shaw R, Drozdowska B, Taylor-Rowan M, Elliott E, Cuthbertson G, Stott DJ (2019). Delirium in an acute stroke setting, occurrence, and risk factors. Stroke.

[41] Haley MN, Casey P, Kane RY, Darzins P, Lawler K (2019). Delirium management: let's get physical? A systematic review and meta-analysis. Australas J Age.

[42] Bernhardt J, Churilov L, Ellery F, Collier J, Chamberlain J, Langhorne P (2016). Prespecified dose-response analysis for a very early rehabilitation trial (AVERT). Neurology.

[43] Shi Q, Presutti R, Selchen D, Saposnik G (2012). Delirium in acute stroke: a systematic review and meta-analysis. Stroke.

[44] Dziegielewski C, Skead C, Canturk T, Webber C, Fernando SM, Thompson LH (2021). Delirium and associated length of stay and costs in critically ill patients. Crit Care Res Pract.

[45] Green S, Reivonen S, Rutter LM, Nouzova E, Duncan N, Clarke C (2018). Investigating speech and language impairments in delirium: a preliminary case-control study. PLoS ONE.

[46] Romoli M, Eusebi P, Forlivesi S, Gentile M, Giammello F, Piccolo L (2021). Stroke network performance during the first COVID-19 pandemic stage: a meta-analysis based on stroke network models. Int J Stroke.

[47] Pabón-Martínez BA, Rodríguez-Pulido LI, Henao-Castaño AM (2021) The family in preventing delirium in the intensive care unit: scoping review. Enferm Intensiva (Engl Ed) 10.1016/j.enfie.2021.01.003 35144905

