# Supplementary material for: Delirium on stroke units: a prospective, multicentric quality-improvement project
Source: J Neurol. 2022 Feb 14;269(7):3735–44. doi: 10.1007/s00415-022-11000-6 (PMC9217833; doi:10.1007/s00415-022-11000-6)
Supplement: Supplementary file 1 — Supplementary file1 (DOCX 69 KB) [file 415_2022_11000_MOESM1_ESM.docx]

**Supplement**

**Delirium on stroke units: a prospective, multicentric quality improvement project**

Dr. rer. hum. biol. Peter Nydahl, RN BScN MScN^1^, Friederike Baumgarte^2^, Prof. Dr. Daniela Berg^3^, Dr. phil. Manuela Bergjan^4^, Dr. phil. Christoph Borzikowsky^5^, Dr. med. Christiana Franke^6^, Diana Green^2^, Anisa Hannig^7^, Prof. Dr. Hans Christian Hansen^7^, Armin Hauss, MScN^4^, Uta Hansen, BScN^8^, Rahel Istel^2^, Norma Krämer, M.A.^7^, Karita Krause^2^, Renée Lohrmann, BSc^4^, Mohammad Mohammadzadeh-Vazifeh^7^, Univ.-Prof. Dr. Dr. h.c. Jürgen Osterbrink^9,10^, PD Dr. Frederick Palm^11^, Telse Petersen^2^, Bernd Schöller, RN^11^, Prof. Dr. med. Henning Stolze^8^, Max Zilezinski, MSc^4,12^, Dr. med Johannes Meyne^3^, PD Dr. med. Dipl.-Psych. Nils G. Margraf^3^

1. Nursing Science and development, Department of Anesthesiology and Intensive Care Medicine, University Hospital of Schleswig-Holstein, Kiel, Germany
2. Christian Albrechts University Kiel, Germany
3. Department of Neurology, University Hospital of Schleswig-Holstein, Kiel, Germany
4. Business Division Nursing Directorate, Nursing Science, Charité – Universitätsmedizin Berlin, corporate member of Freie Universität Berlin, Humboldt-Universität zu Berlin and Berlin Institute of Health, Berlin, Germany
5. Institute of Medical Informatics und Statistics, Kiel University, University Hospital Schleswig-Holstein, Kiel, Germany
6. Department of Neurology, Charité – Universitätsmedizin Berlin, corporate member of Freie Universität Berlin, Humboldt-Universität zu Berlin and Berlin Institute of Health, Berlin, Germany
7. Department of Neurology, Friedrich-Ebert-Krankenhaus, Neumünster, Germany
8. Department of Neurology, Diako Flensburg, Germany
9. Institut für Pflegewissenschaft und-praxis; Paracelsus Medizinische Privatuniversität, Salzburg, Austria
10. Brooks College of Health, University of North Florida, Jacksonville, USA
11. Department of Neurology, Heliosklinikum Schleswig, Germany
12. University Medicine Halle (Saale), Health Service Research Working Group | Acute Care, Department of Internal Medicine, Faculty of Medicine, Martin-Luther-University Halle-Wittenberg, Halle

**Content**

**Table E1** Delirium Management in 18 delirious stroke patients

**Table E2** Comparison between centers in sociodemographic data

**Table E3** Sociodemographic data in non-delirious vs delirious patients

**Table E4** Outcome in non-delirious vs delirious patients

**Table E5** Outcome between centers

**Table E6** Delirium outcome between centers

**Figure E1** Comparison of difference in modified Rankin Scale at admission – discharge in non-/delirious patients with modified Rankin Scale ≥ 3 at admission

**Table E7**: SQUIRE

**Table E1: Delirium Management in 18 delirious stroke patients**

| **Multiple precipitants identified (n=96)** | **Precipitants** | **Treatments** | **Treatments delivered (n=65)** |
| --- | --- | --- | --- |
| **9 (9.4)** | **Acute Illness** |  | **12 (18.5)** |
| 2 (2.1) | Shock (MAP < 65 mmHg > 15 Min) | Stabilisation (position, volume, vasopressors) till MAP > 65 mmHg | 2 (3.1) |
| 2 (2.1) | Hypoxia (sO2 < 90% > 15 Min) | Administer O_2_ till sO_2_ > 94%, facilitate patient’s breathing | 3 (4.6) |
| 5 (5.2) | Infection (breathing frequency ≥ 22/min., GCS < 15, blood pressure ≤ 100 mmHg, CRP ≥ 5, temp. ≥ 37.5) | Antipyretics ≥ 37.5 C., microbiological stewardship & antibiotics ≥ 38.0 C., consider change of lines & tubes | 5 (7.7) |
| 0 (0) | Metabolic disturbances (e.g. low natrium) | Blood samples, correction of disturbance | 2 (3.1) |
| **61 (63.5)** | **Environmental factors** |  | **33 (50.8)** |
| 16 (16.7) | Lines & Tubes | Bundle: hygienic rules, daily reflection of necessity, evaluate discomfort by wrong position, consider change or removal | 8 (12.3) |
| 11 (11.4) | Noise | Reduction of noise, adaption of alarms, ear plugs, close doors, early change of syringes/infusions without alarms, evaluate white noise/music application | 5 (7.7) |
| 8 (8.3) | Lack of sleep | Consideration of personal sleep habits, ask for causes of sleep disorders & revise, if possible, listen & comfort, reduce light and noise, consider mobilization during the day and evening, consider video-call with loved ones to say good night | 5 (7.7) |
| 12 (12.5) | Immobility | Mobilization 6:00 a.m. till 11:00 p.m. | 9 (13.8) |
| 14 (14.5) | Lack of social encounters | Offer talks, listen & comfort, integrate families | 5 (7.7) |
| **12 (12.5)** | **Medication** |  | **10 (15.4)** |
| 5 (5.2) | Polypharmacy ≥ 10 drugs/day | Evaluate, adapt, consider geriatrics’/pharmacists’ counsel | 5 (7.7) |
| 3 (3.1) | Withdrawal | Adapt and creep out. In case of alcohol withdrawal, use SOP | 2 (3.1) |
| 1 (1) | Benzodiazepines | Avoid (only as rescue drug) | 1 (1.5) |
| 1 (1) | Anticholinergics | Avoid (only as rescue drug) | 0 (0) |
| 0 (0) | Opioids | Pain Counsel | 1 (1.5) |
| 2 (2.1) | Pain ≥ 3 Numeric Rating Scale | Analgesia | 1 (1.5) |
| **14 (14.6)** | **Neurology** |  | **10 (15.4)** |
| 11 (11.4) | Cerebral ischemia or stroke | Bundle re-perfusion: consider endovascular or lyse therapy, monitoring blood pressure & neurological symptoms, avoid too long upright position, consider increased brain pressure | 5 (7.7) |
| 3 (3.1) | Haemorrhagic stroke | Bundle intracranial bleeding: patient education, upright position 30^0^, monitoring of blood pressure and neurological symptoms, analgesia, consider increased brain pressure | 2 (3.1) |
| 0 (0) | Epilepsy | Antiepileptic drugs, avoid complications | 3 (4.6) |
| 0 (0) | Encephalitis, Meningitis | Antibiotics/antivirals, patient education, sufficient analgesia, possibly intracranial pressure therapy, | 0 () |
| 0 (0) | Others | Others | 0 (0) |

Data are reported as absolute and relative frequencies (percentages). Percentages may not sum up to 100 due to rounding.

**Table E2** Comparison between centers in sociodemographic data

| Item | All  (n=475) | 1  (n=116) | 2  (n=100) | 3  (n=52) | 4  (n=84) | 5  (n=123) | p |
| --- | --- | --- | --- | --- | --- | --- | --- |
| Preadmission |  |  |  |  |  |  |  |
| Most frequent age decile (%) | <80 (28.5%) | <80 (33.9%) | <80 (30.3%) | <60/80 (25%) | <90 (30.5) | <80 (28.7) | 0.404 |
| Female gender (n, %) | 194 (43.6) | 50 (43.5) | 40 (45.5) | 21 (42) | 31 (43.7) | 52 (43) | 0.996 |
| Living in nursing home | 22 (4.6) | 11 (9.6) | 3 (3.2) | 0 (0) | 2 (2.9) | 6 (4.9) | 0.068 |
| Pre-existing depression | 11 (2.3) | 4 (3.4) | 1 (1) | 0 (0) | 1 (1.2) | 5 (4.1) | 0.431 |
| Pre-existing dementia | 16 (3.3) | 8 (6.8) | 0 (0) | 0 (0) | 1 (1.2) | 7 (5.7) | 0.407 |
| Modified Rankin Scale | 0 (0-1) | 0 (0-1) | 0 (0-0) | 0 (0-0) | - | 0 (0-1) | 0.008^ns^ |
| Admission |  |  |  |  |  |  |  |
| C-reactive protein (mg/l) | 1.7 (0.6-4.4) | 2.4 (1.1-7) | 3 (1-7.1) | 1 (0-3) | 2.6 (0.5-8.4) | 2.9 (1.1-5.1) | 0.009 |
| Natrium (mmol/l) | 139 (137-141) | 140 (137-142) | 139 (138-142) | 140 (138-142) | 140 (137-141) | 139 (136-141) | 0.064 |
| Body Mass Index (kg/m2) | 26.2 (23.6-29.4) | 26.3 (22.7-29.4) | 23.9 (23.4-25) | 25.4 (23.4-29.5) | 26.7 (23.4-32.7) | 26.2 (24.5-28.9) | 0.398 |
| Neurological state |  |  |  |  |  |  |  |
| NIHSS | 2 (0-4) | 1 (0-4) | 2 (1-5) | 2 (0-2.5) | 1 (0-2) | 2 (1-6.7) | 0.141 |
| Modified Rankin Scale | 2 (1-3) | 1 (0-3) | 2 (1-3) | 2 (0-2) | 2 (1-3) | 2 (1-4) | 0.00005 |
| Primary diagnosis |  |  |  |  |  |  | 0.339 |
| Ischemic stroke | 319 (69.5) | 75 (64.7) | 64 (66.7) | 33 (64.7) | 48 (64.9) | 99 (81.1) |  |
| Trans ischemic Attack (TIA) | 93 (20.3) | 26 (22.4) | 24 (25) | 10 (19.6) | 14 (18.9) | 19 (15.6) |  |
| Hemorrhagic stroke | 13 (2.7) | 4 (3.4) | 2 (2) | 1 (1.9) | 3 (3.6) | 3 (2.4) |  |
| Cerebral venous sinus thrombosis | 1 (0.2) | 0 (0) | 0 (0) | 0 (0) | 0 (0) | 1 (0.8) |  |
| Epilepsy | 3 (0.6%) | 3 (2.6) | 0 (0) | 0 (0) | 0 (0) | 0 (0) |  |
| Migraine | 4 (0.8) | 0 (0) | 2 (2) | 2 (3.8) | 0 (0) | 0 (0) |  |
| Others | 26 (5.5) | 8 (6.9) | 4 (4) | 5 (9.6) | 9 (10.7) | 0 (0) |  |
| Interventions |  |  |  |  |  |  |  |
| Intravenous thrombolysis | 47 (9.9) | 14 (12.1) | 9 (9) | 6 (11.5) | 2 (2.4) | 16 (13) | 0.111 |
| Endovascular thrombectomy | 22 (4.6) | 4 (3.4) | 2 (2) | 3 (5.8) | 0 (0) | 13 (10.6) | 0.003 |

Abbreviations: ns non-significant after Bonferroni correction to p_adjust_ = 0.0005

Data reported in n (%) or median (Interquartile range)

**Table E3** Sociodemographic data in non-delirious vs delirious patients

| **Item** | **All (n=475)** | **Not delirious (n=430)** | **Delirious (n=45)** | **p** |
| --- | --- | --- | --- | --- |
| **Preadmission** |  |  |  |  |
| Most frequent age decile in years (%) | <80 (28.5) | <80 (28.6) | <90 (41.9) | 0.080 |
| Female gender (n, %) | 194 (43.6) | 177 (43.8) | 17 (41.5) | 0.869 |
| Living at home | 422 (95) | 387 (96.5) | 35 (81.4) | 0.00046 |
| Living in nursing home | 22 (5) | 14 (3.5) | 8 (18.6) |  |
| Pre-existing depression | 11 (2.3) | 8 (1.9) | 3 (6.7) | 0.076 |
| Pre-existing dementia | 16 (3.4) | 10 (2.3) | 6 (13.3) | 0.002^ns^ |
| Modified Rankin Scale | 0 (0-1) | 0 (0-0) | 0 (0-3) | 0.001^ns^ |
| **Admission** |  |  |  |  |
| C-reactive protein (mg/l) | 2.5 (1-6) | 2.2 (1-5.6) | 4.9 (2-10.1) | 0.001^ns^ |
| Natrium (mmol/l) | 139 (137-141) | 139 (137-141) | 139 (135-142) | 0.762 |
| Body Mass Index (kg/m2) | 26.2 (23.6-29.4) | 26.2 (23.7-29.4) | 25.7 (22.6-30.3) | 0.612 |
| **Neurological state** |  |  |  |  |
| NIHSS 0 (no stroke symptoms) | 121 (28.3) | 118 (30.4) | 3 (7.5) | <0.0001 |
| NIHSS 1-4 (minor stroke) | 211 (49.3) | 194 (50) | 17 (42.5) |  |
| NIHSS 5-42 (moderate to severe stroke) | 96 (22.4) | 76 (19.6) | 20 (50) |  |
| Modified Rankin Scale | 2 (1-3) | 2 (1-3) | 4 (3-5) | <0.0001 |
| **Primary diagnosis** |  |  |  | 0.590 |
| Ischemic stroke | 319 (69.5) | 286 (66.5) | 33 (76.7) |  |
| Trans ischemic Attack (TIA) | 93 (20.3) | 89 (21.4) | 4 (9.3) |  |
| Hemorrhagic stroke | 13 (2.8) | 9 (2.2) | 4 (9.3) |  |
| Cerebral venous sinus thrombosis | 1 (0.2) | 1 (0.2) | 0 (0) |  |
| Epilepsy | 3 (0.7) | 3 (0.7) | 0 (0) |  |
| Migraine | 4 (0.9) | 4 (1) | 0 (0) |  |
| Others | 26 (5.7) | 24 (5.8) | 2 (4.7) |  |
| **Interventions** |  |  |  |  |
| Intravenous thrombolysis | 47 (9.9) | 36 (8.4) | 11 (24.4) | 0.002^ns^ |
| Endovascular thrombectomy | 22 (4.6) | 19 (4.4) | 3 (6.7) | 0.453 |

Abbreviations: ns non-significant after Bonferroni correction to p_adjust_ = 0.0005

Data reported in n (%) or median (Interquartile range)

**Table E4** Outcome in non-delirious vs delirious patients

| Item | All (n=475) | Not delirious (n=430) | Delirious (n=45) | p |
| --- | --- | --- | --- | --- |
| Modified Rankin Scale at discharge | 1 (0-3) | 1 (0-2) | 4 (3-5) | <0.0001 |
| Length of stay in Stroke Unit (days) | 3 (2-4) | 3 (2-4) | 5 (3.5-7) | <0.0001 |
| Length of stay in hospital (days) | 6 (4-9) | 5.5 (4-8) | 11 (5.7-14.2) | <0.0001 |
| Mortality in Stroke Unit | 3 (0.6) | 1 (0.2) | 2 (4.4) | 0.008^ns^ |
| Mortality in hospital | 6 (1.3) | 2 (0.5) | 4 (8.9) | 0.0009^ns^ |
| Complications* | 51 | 16 | 35 | 0.0001 |
| Dehydration | 8 (1.7) | 3 (0.7) | 5 (11.1) |  |
| Undernutrition | 5 (1) | 1 (0.2) | 4 (8.9) |  |
| Pressure sore | 1 (0.2) | 0 (0) | 1 (2.2) |  |
| Fall | 6 (1.3) | 3 (7) | 3 (6.7) |  |
| Immobility | 12 (2.7) | 6 (1.4) | 6 (13.3) |  |
| Restraints | 4 (0.8) | 0 (0) | 4 (8.9) |  |
| Decreasing compliance | 7 (1.5) | 2 (0.5) | 5 (11.1) |  |
| Removal of lines | 8 (1.7) | 1 (0.2) | 7 (15.6) |  |
| Discharge localization |  |  |  |  |
| Home | 271 (57.1) | 262 (60.9) | 9 (20) | <0.0001 |
| Rehabilitation facility | 56 (11.8) | 46 (10.7) | 10 (22.2) |  |
| Other hospital | 20 (4.2) | 13 (3) | 7 (15.6) |  |
| Nursing home | 17 (3.6) | 11 (2.6) | 6 (13.3) |  |
| Missing information | 111 (23.4) | 98 (22.8) | 13 (28.9) |  |

Data reported in n (%) or median (Interquartile range)

*patients could have more than one complication

Abbreviations: ns non-significant after Bonferroni correction to p_adjust_ = 0.0005

**Table E5** Outcome between centers

| Item | All  (n=475) | 1  (n=116) | 2  (n=100) | 3  (n=52) | 4  (n=84) | 5  (n=123) | p |
| --- | --- | --- | --- | --- | --- | --- | --- |
| Modified Rankin Scale at discharge | 1 (0-2.2) | 1 (0-2) | 1 (0-2,7) | 0 (0-1) | 1 (0-3) | 2 (0-4) | 0.00005 |
| Length of stay in Stroke Unit (days) | 3 (2-4) | 3 (3-4) | 2 (2-3) | 2 (2-3) | 3 (2-4) | 3 (2-4) | <0.0001 |
| Length of stay in hospital (days) | 6 (4-9) | 5 (3-8) | 6 (5-9) | 8 (6-9.2) | 5 (3-8) | 6 (4-9) | <0.0001 |
| Mortality in Stroke Unit | 3 (0.6) | 1 (0.9) | 0 (0) | 0 (0) | 1 (1.2) | 1 (0.8) | n/a |
| Mortality in hospital | 6 (1.3) | 3 (2.6) | 1 (1) | 2 (3.8) | 0 (0) | 0 (0) | n/a |
| Discharge localization |  |  |  |  |  |  | 0.001^ns^ |
| Home | 271 (57.1) | 86 (74.1) | 60 (60) | 37 (71.2) | - | 88 (71.5) |  |
| Rehabilitation facility | 56 (11.8) | 15 (12.9) | 16 (16) | 11 (21.2) | 1 (1.2) | 13 (10.6) |  |
| Other hospital | 20 (4.2) | 0 (0) | 12 (12) | 1 (1.9) | 1 (1.2) | 6 (4.9) |  |
| Nursing home | 17 (3.6) | 9 (7.8) | 3 (3) | 0 (0) | - | 5 (4.1) |  |
| Missing | 111 (23.4) | 6 (5.2) | 9 (9) | 3 (5.8) | 82 (97.6) | 11 (8.9) |  |

Data reported in n (%) or median (Interquartile range)

Abbreviations: ns non-significant after Bonferroni correction to p_adjust_ = 0.0005

**Table E6** Delirium outcome between centers

| Item | All  (n=475) | 1  (n=116) | 2  (n=100) | 3  (n=52) | 4  (n=84) | 5  (n=123) | p |
| --- | --- | --- | --- | --- | --- | --- | --- |
| Delirium incidence | 45 (9.5) | 15 (12.9) | 7 (7) | 3 (5.8) | 7 (8.3) | 13 (10.6) | 0.484 |
| Before | 21 (8.4) | 7 (10.9) | 1 (1.9) | 2 (7.4) | 4 (7.7) | 7 (12.5) | 0.322 |
| After | 24 (10.7) | 8 (15.4) | 6 (12.5) | 1 (4) | 3 (9.4) | 6 (9) | 0.592 |
| Median Nu-DESC | 3.2 (2.3-4.3) | 3.4 (2.8-5.8) | 2 (1-3) | 2.5 (2.5-2.5) | 3 (2-3.3) | 4 (2.5-4.8) | 0.074 |
| Before | 3.5 (2.6-4.7) | 3.5 (3.3-4.2) | 3.2 (3.2-3.2) | 3.1 (2.5-3.1) | 2.7 (2-5.3) | 4.5 (2.7-5) | 0.621 |
| After | 3 (2.2-4) | 3.1 (2.5-6) | 2 (0.7-3.4) | 2.5 (2.5-2.5) | 3 (2-3) | 3.9 (2.3-4.2) | 0.214 |

Abbreviations: Nu-DESC Nursing Delirium Screening Scale

Data reported in n (%) or median (Interquartile range)

Figure E1: Comparison of difference in modified Rankin Scale at admission – discharge in non-/delirious patients with modified Rankin Scale ≥ 3 at admission


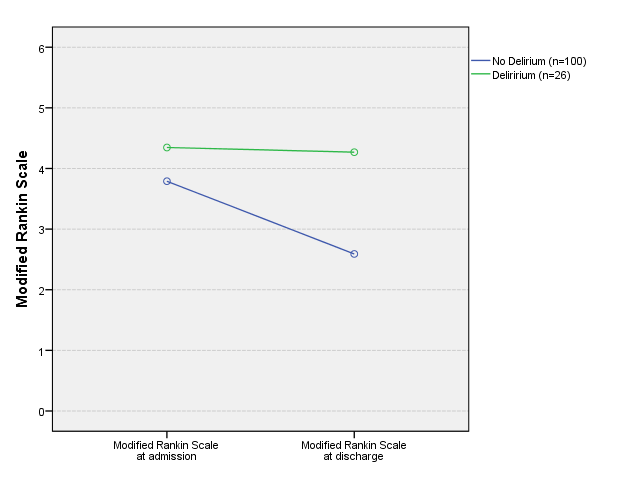


**Table E7 SQUIRE**

Revised Standards for Quality Improvement Reporting Excellence (SQUIRE 2.0) September 15, 2015

| **Text Section and Item Name** | **Section or Item Description** | Page |
| --- | --- | --- |
| **Title and Abstract** |  |  |
| **1. Title** | Indicate that the manuscript concerns an [initiative](#_bookmark6) to improve healthcare (broadly defined to include the quality, safety, effectiveness, patient- centeredness, timeliness, cost, efficiency, and equity of healthcare) | 1 |
| **2. Abstract** | 1. Provide adequate information to aid in searching and indexing 2. Summarize all key information from various sections of the text using the abstract format of the intended publication or a structured summary such as: background, local [problem,](#_bookmark10) methods, interventions, results, conclusions | 1-2 |
| **Introduction** | *Why did you start?* |  |
| **3. Problem Description** | Nature and significance of the local [problem](#_bookmark10) | 3 |
| **4. Available knowledge** | Summary of what is currently known about the [problem,](#_bookmark10) including relevant previous studies | 3-4 |
| **5. Rationale** | Informal or formal frameworks, models, concepts, and/or [theories](#_bookmark14) used to explain the [problem,](#_bookmark10) any reasons or [assumptions](#_bookmark0) that were used to develop the [intervention(s)](#_bookmark8), and reasons why the [intervention(s)](#_bookmark8) was expected to work | 4-5 |
| **6. Specific aims** | Purpose of the project and of this report | 4 |
| **Methods** | What did you do? |  |
| **7.** [**Context**](#_bookmark1) | Contextual elements considered important at the outset of introducing the [intervention(s)](#_bookmark8) | 5 |
| **8. Intervention(s)** | 1. Description of the [intervention(s)](#_bookmark8) in sufficient detail that others could reproduce it   Specifics of the team involved in the work | 5 |
| **9. Study of the Intervention(s)** | 1. Approach chosen for assessing the impact of the [intervention(s)](#_bookmark8)   Approach used to establish whether the observed outcomes were due to the [intervention(s)](#_bookmark8) | 6 |
| **10. Measures** | 1. Measures chosen for studying [processes](#_bookmark11) and outcomes of the [intervention(s)](#_bookmark8), including rationale for choosing them, their operational definitions, and their validity and reliability 2. Description of the approach to the ongoing assessment of contextual elements that contributed to the success, failure, efficiency, and cost   Methods employed for assessing completeness and accuracy of data | 6 |
| **11. Analysis** | 1. Qualitative and quantitative methods used to draw [inferences](#_bookmark5) from the data   Methods for understanding variation within the data, including the effects of time as a variable | 7 |
| **12. Ethical**  **Considerations** | [Ethical aspects](#_bookmark2) of implementing and studying the [intervention(s)](#_bookmark8) and how they were addressed, including, but not limited to, formal ethics review and potential conflict(s) of interest | 4 |
| **Results** | *What did you find?* |  |
| **13. Results** | 1. Initial steps of the [intervention(s)](#_bookmark8) and their evolution over time (*e.g.*, time-line diagram, flow chart, or table), including modifications made to the intervention during the project 2. Details of the [process](#_bookmark11) measures and outcome 3. Contextual elements that interacted with the [intervention(s)](#_bookmark8) 4. Observed associations between outcomes, interventions, and relevant contextual elements 5. Unintended consequences such as unexpected benefits, problems, failures, or costs associated with the [intervention(s).](#_bookmark8)   Details about missing data | 8-10 |
| **Discussion** | *What does it mean?* |  |
| **14. Summary** | 1. Key findings, including relevance to the [rationale](#_bookmark12) and specific aims   Particular strengths of the project | 11 |
| **15. Interpretation** | 1. Nature of the association between the [intervention(s)](#_bookmark8) and the outcomes 2. Comparison of results with findings from other publications 3. Impact of the project on people and [systems](#_bookmark13) 4. Reasons for any differences between observed and anticipated outcomes, including the influence of [context](#_bookmark1)   Costs and strategic trade-offs, including [opportunity costs](#_bookmark9) | 11-12 |
| **16. Limitations** | 1. Limits to the [generalizability](#_bookmark3) of the work 2. Factors that might have limited [internal validity](#_bookmark7) such as confounding, bias, or imprecision in the design, methods, measurement, or analysis   Efforts made to minimize and adjust for limitations | 12-13 |
| **17. Conclusions** | 1. Usefulness of the work 2. Sustainability 3. Potential for spread to other [contexts](#_bookmark1) 4. Implications for practice and for further study in the field   Suggested next steps | 13 |
| **Other information** |  |  |
| **Funding** | Sources of funding that supported this work. Role, if any, of the funding organization in the design, implementation, interpretation, and reporting | Detail page |

Ogrinc, G., Davies, L., Goodman, D., Batalden, P., Davidoff, F., & Stevens, D. (2016). SQUIRE 2.0 (Standards for QUality Improvement Reporting Excellence): revised publication guidelines from a detailed consensus process. *BMJ quality & safety*, *25*(12), 986–992. https://doi.org/10.1136/bmjqs-2015-004411
